# 249. Characteristics and outcomes of cryptococcosis among patients with COVID-19 compared to non-COVID-19 controls

**DOI:** 10.1093/ofid/ofac492.327

**Published:** 2022-12-15

**Authors:** Daniel B Chastain, Vanessa Kung, Sahand Golpayegany, Brittany T Jackson, Carlos Franco-Paredes, George R Thompson, Andrés F Henao-Martínez

**Affiliations:** University of Georgia College of Pharmacy, Albany, Georgia; University of Colorado, Denver, Colorado; University of Georgia, Marietta, Georgia; The Mount Sinai Hospital, New York, New York; University of Colorado Anschutz Medical Campus, Aurora, Colorado; University of California Davis Medical Center, Sacramento, California; University of Colorado Anschutz Medical Campus, Aurora, Colorado

## Abstract

**Background:**

It remains unclear if there is an association between COVID-19 and cryptococcosis. The purpose of this study was to compare demographic characteristics and outcomes of cryptococcosis between patients with COVID-19 to non-COVID-19 controls.

**Methods:**

Patients 18 years and older with cryptococcosis were identified from TriNetX, a global federated research network, and separated into two cohorts based on a diagnosis of COVID-19 within 3 months prior to the index diagnosis of cryptococcosis. The primary outcome was the percent mortality in each group. The secondary outcomes included the proportion of patients in each group who had underlying comorbidities, received immunosuppressive medications, or required hospitalization or ICU admission. Propensity score matching was performed to control for differences between groups based on demographics and comorbidities. Odds ratios (ORs) with 95% confidence intervals (CIs) were calculated for outcomes, with p < 0.05 as the cut off for statistical significance.

**Results:**

A total of 6252 patients with cryptococcosis were included, of which 4.5% (n=283) had COVID-19 prior to diagnosis of cryptococcosis. Mortality was similar between patients with and without COVID-19 (13% vs 10%, p=0.075). Patients with cryptococcosis and previous COVID-19 were older (55.2 ± 14.5 years vs 52 ± 15.2 years, p=0.0005) and more likely to be non-Hispanic (73% vs 65%, p=0.0049). More patients with COVID-19 had a history of transplant (30% vs 13%, p < 0.0001), malignancy (37% vs 21%, p < 0.0001), and diabetes (35% vs 19%, p < 0.0001), but not HIV (29% vs 31%, p=0.5482). Prednisone and dexamethasone use were higher among patients with previous COVID-19 (32% vs 15%, p < 0.0001 and 17% vs 7%, p < 0.0001, respectively). Hospitalization rates were similar (54% vs 57%, p=0.278), but more patients with COVID-19 required ICU admission (19% vs 11%, p < 0.0001). In propensity score-matched analysis, patients with COVID-19 remained at higher odds of ICU admission (OR 1.85, 95% CI 1.15-2.97, p=0.010), but lower odds of hospitalization (OR 0.57, 95% CI 0.41-0.81, p=0.001).

**Conclusion:**

Patients with COVID-19 who developed cryptococcosis had higher rates of comorbidities, corticosteroid use, and ICU admission but did not experience higher mortality compared to non-COVID-19 controls.

**Disclosures:**

**George R. Thompson, III, MD**, Amplyx: Advisor/Consultant|Amplyx: Grant/Research Support|Astellas: Advisor/Consultant|Astellas: Grant/Research Support|Cidara: Advisor/Consultant|Cidara: Grant/Research Support|F2G: Advisor/Consultant|F2G: Grant/Research Support|Merck: Grant/Research Support|Pfizer: DSMB|Scynexis: Advisor/Consultant|Scynexis: Grant/Research Support.

